# Navigated Transcranial Magnetic Stimulation (nTMS): From Functional Brain Mapping to Clinical Applications in Neurosurgery and Neurology

**DOI:** 10.3390/biomedicines14051152

**Published:** 2026-05-19

**Authors:** Marcin Karol Setlak, Bartłomiej Błaszczyk, Maciej Wojtacha, Adam Rudnik

**Affiliations:** Department of Neurosurgery, University Clinical Center, Faculty of Medical Sciences in Katowice, Medical University of Silesia, 40-752 Katowice, Poland

**Keywords:** navigated transcranial magnetic stimulation, neurosurgery, functional mapping, cortical excitability, motor evoked potentials

## Abstract

**Introduction:** Navigated transcranial magnetic stimulation (nTMS) is an advanced, noninvasive method for stimulation-based functional brain mapping. Its main clinical value in neurosurgery lies in preoperative identification of eloquent cortical areas and the integration of functional information into neuronavigation-based surgical planning. **State of the Art:** This narrative review with a structured literature search summarizes the historical and technical foundations of TMS/nTMS, but primarily focuses on neurosurgical applications, including motor and language mapping, comparison with functional MRI and direct cortical stimulation, safety considerations, and practical limitations. Broader neurological and therapeutic applications are discussed as contextual extensions rather than as a comprehensive disease-specific review. **Clinical Implications:** Current evidence is strongest for preoperative motor mapping in patients with tumors located in or near the motor–eloquent cortex. Language mapping, neurological diagnostics, and therapeutic repetitive TMS (rTMS) applications remain more heterogeneous and require careful interpretation according to the level of evidence, protocol standardization, and patient selection. **Future Directions:** Further multicenter studies, standardized mapping protocols, integration with advanced imaging and tractography, and health-system implementation strategies are needed to define the optimal role of nTMS in personalized neurosurgical and neurological care.

## 1. Introduction

Mapping eloquent cortical areas remains a central challenge in neurosurgery, particularly in patients undergoing resection of brain tumors located in or near motor, language, or associative cortical regions [1,2]. The modern goal of neuro-oncological surgery is not simply lesion removal, but maximal safe resection: achieving the greatest feasible extent of resection while preserving neurological function and quality of life [1,3]. Intraoperative neuromonitoring (IONM) and direct cortical or subcortical stimulation remain essential tools during surgery [1,2], but they do not provide a complete preoperative functional map before the craniotomy has been planned [4,5].

Functional magnetic resonance imaging (fMRI), magnetoencephalography (MEG), tractography, and other imaging-based techniques have long been used to support preoperative planning [6]. However, these approaches have important limitations, including dependence on patient cooperation, susceptibility to neurovascular uncoupling in tumor tissue, variable spatial accuracy, and limited direct electrophysiological validation [6,7]. Navigated transcranial magnetic stimulation (nTMS) addresses part of this gap by combining noninvasive cortical stimulation with individual structural imaging and real-time neuronavigation [4,8].

nTMS is clinically distinct from conventional transcranial magnetic stimulation (TMS). While conventional TMS allows noninvasive stimulation of cortical tissue, nTMS links each stimulation site to the patient’s own magnetic resonance imaging dataset, thereby enabling spatial localization of electrophysiologically relevant cortical points [8,9]. This is particularly important in neuro-oncology, where mass effect, cortical displacement, edema, prior surgery, or tumor-related reorganization may reduce the reliability of anatomical assumptions [4,9]. By connecting electrophysiological responses to patient-specific coordinates, nTMS provides a preoperative functional map that can be incorporated into neuronavigation and used to guide surgical strategy [4,10].

This review primarily focuses on the role of nTMS in neurosurgical preoperative mapping and perioperative decision-making. Broader diagnostic and therapeutic applications of TMS in neurology and neurorehabilitation are discussed as contextual extensions of the method rather than as a comprehensive review of all TMS-based interventions. This scope reflects the current clinical relevance of nTMS as an adjunct to fMRI, tractography, neuronavigation, IONM, and direct cortical stimulation in patients undergoing surgery for lesions located near eloquent cortical areas [1,4,5].

## 2. Methods: Literature Search Strategy

This article was designed as a narrative review with a structured literature search rather than as a systematic review or meta-analysis. The aim was to synthesize clinically relevant evidence on navigated transcranial magnetic stimulation (nTMS), with particular emphasis on preoperative functional mapping and neurosurgical workflow integration.

A literature search was performed using PubMed/MEDLINE and Google Scholar, with additional targeted screening of references cited in key guidelines, systematic reviews, meta-analyses, and clinically relevant original studies. The following search terms were used alone and in combination: “navigated transcranial magnetic stimulation”, “nTMS”, “preoperative mapping”, “motor mapping”, “language mapping”, “brain tumor”, “glioma”, “eloquent cortex”, “direct cortical stimulation”, “direct electrical stimulation”, “functional MRI”, “neuronavigation”, “tractography”, “repetitive transcranial magnetic stimulation”, “TMS safety”, and “seizure risk”. The literature search was last updated in March 2026 and included publications available up to that date. No strict date restriction was applied; however, recent systematic reviews, meta-analyses, expert guidelines, and clinically relevant studies published within the last decade were preferentially considered when available.

Priority was given to systematic reviews, meta-analyses, expert guidelines, prospective and retrospective clinical studies, and technical reports directly relevant to nTMS in neurosurgical practice. Studies were selected if they addressed technical or neurophysiological principles of TMS/nTMS, preoperative motor or language mapping, comparison with functional MRI or direct cortical/electrical stimulation, integration with neuronavigation or tractography, safety considerations, or clinical implementation barriers. Broader neurological and therapeutic applications of TMS were included only when they provided context for clinical interpretation or guideline-based use. Because this was not a systematic review, no formal risk-of-bias assessment, PRISMA-based study selection process, or quantitative evidence synthesis was performed.

## 3. Historical, Technical, and Neurophysiological Foundations of TMS and nTMS

### 3.1. Brief Historical Development

The development of TMS rests on a long electrophysiological tradition. Luigi Galvani’s experiments on electrically induced muscle contraction and Michael Faraday’s discovery of electromagnetic induction provided the conceptual foundations for later noninvasive stimulation techniques [11,12,13].

In 1980, Merton and Morton demonstrated transcranial electrical stimulation of the human motor cortex, but the method was painful because of scalp and facial muscle activation [14].

In 1985, Anthony Barker and colleagues at the University of Sheffield achieved the first painless, noninvasive stimulation of the motor cortex using a rapidly changing magnetic field generated by a stimulation coil placed over the scalp. This experiment, which produced measurable motor responses in the contralateral hand, marked the birth of transcranial magnetic stimulation (TMS) [15].

However, conventional TMS alone did not provide the spatial precision required for neurosurgical planning. The integration of TMS with neuronavigation systems enabled reproducible stimulation of patient-specific cortical targets and gave rise to nTMS as a clinically useful technique for preoperative functional mapping [4,8,9].

### 3.2. Technical Principles and Neuronavigation

A TMS stimulator contains a capacitor, a high-voltage charging system, and a rapidly switching circuit that discharges current through a stimulation coil. The resulting rapidly changing magnetic field induces an electric field in the underlying cortical tissue. Modern stimulators allow adjustment of pulse waveform, pulse intensity, frequency, train duration, and inter-train intervals, which enables single-pulse mapping as well as repetitive stimulation protocols [16,17].

Coil geometry strongly influences the focality and depth of stimulation. Figure-of-eight coils remain the standard for relatively focal cortical stimulation, especially in motor mapping, whereas double-cone, H-coil, and other geometries trade focality for greater field penetration or broader stimulation volumes [18,19,20]. This technical compromise is clinically important: even with neuronavigation, the stimulated volume is not a mathematical point, and stimulation of small or deeply located structures remains limited by coil size, coil–cortex distance, head anatomy, and induced electric-field geometry [19,20].

Neuronavigated systems use the patient’s structural MRI and tracking of the stimulation coil in relation to the head to display the predicted stimulation site and coil orientation in real time. This improves reproducibility compared with scalp-based or anatomical landmark-based approaches [4,8,21]. In neurosurgical applications, nTMS-derived functional points can be exported to neuronavigation platforms and combined with structural MRI, fMRI, and diffusion tractography, thereby creating a multimodal surgical planning environment [4,9,10].

### 3.3. Neurophysiological Mechanisms Relevant to Clinical Mapping

TMS induces electric currents within cortical tissue and preferentially activates neuronal elements according to their orientation relative to the induced field. In the motor cortex, stimulation can generate descending volleys through the corticospinal tract, resulting in motor-evoked potentials (MEPs) recorded from contralateral muscles using surface electromyography [17,22]. The resting motor threshold (MT), commonly defined as the minimum stimulation intensity required to elicit an MEP of at least 50 μV in 50% of trials, provides a practical measure of corticospinal excitability and is used to individualize stimulation intensity [17].

TMS does not stimulate all neural tissue equally. Current density decreases with depth, and stimulation is influenced by coil orientation, skull-to-cortex distance, gyral anatomy, and cortical microstructure. Consequently, superficial cortical and cortico-cortical axons are more readily activated than deep subcortical structures [17,19,22]. Pharmacological studies indicate that MT and MEP amplitude reflect partly distinct physiological mechanisms: sodium channel blockers and glutamatergic transmission affect threshold measures, whereas inhibitory and excitatory synaptic mechanisms can modulate MEP amplitude [23].

Modern approaches increasingly interpret TMS not only as a localization tool, but also as a method for probing structure–function relationships. High-resolution mapping and electric-field modeling can link behavioral or electrophysiological responses to modeled cortical stimulation patterns, potentially improving the biological interpretation of stimulation effects and reducing uncertainty related to diffuse field spread [21]. These mechanistic developments are important for future nTMS workflows, but their translation into routine neurosurgical decision-making remains under active investigation [24].

## 4. nTMS in Neurosurgical Preoperative Mapping

### 4.1. Patient Selection and Clinical Indications

The strongest clinical rationale for nTMS is preoperative functional mapping in patients with tumors or other lesions located in or near the eloquent cortex. Typical indications include lesions close to the precentral gyrus, corticospinal tract, opercular regions, inferior frontal gyrus, posterior temporal language regions, insula, angular gyrus, or regions where anatomical displacement makes localization uncertain. nTMS is particularly useful when functional risk must be estimated before deciding on craniotomy size, surgical corridor, need for awake mapping, or the balance between maximal resection and functional preservation [1,4,9,10,25].

nTMS should not be interpreted as a replacement for intraoperative mapping. Direct cortical and subcortical stimulation remain the intraoperative reference standard. The main strength of nTMS is that it provides functional information before surgery, when the operative plan can still be modified. It may also guide where intraoperative stimulation should be most carefully applied and where functional risk is likely to be highest [1,4,5].

### 4.2. Motor Mapping

Motor mapping is the most established neurosurgical application of nTMS. Single-pulse stimulation is used to identify cortical sites that elicit MEPs in selected contralateral muscles. Mapping commonly begins with determination of the motor hotspot and MT, followed by systematic stimulation around the presumed primary motor cortex and adjacent premotor regions. The resulting map can identify cortical representations of the upper limb, lower limb, and facial muscles, and can help estimate the spatial relationship between the lesion, motor cortex, and corticospinal tract [1,9,10,17].

Clinical evidence for motor mapping is stronger than for most other nTMS applications. Observational studies and meta-analyses suggest that preoperative nTMS motor mapping may influence surgical strategy, support smaller and more tailored craniotomies, improve risk stratification, and may be associated with lower rates of permanent postoperative motor deficits and higher rates of gross-total resection in selected motor-eloquent tumors [9,10,25,26,27]. However, the available evidence is still dominated by non-randomized studies, heterogeneous patient cohorts, and variable institutional workflows. Therefore, claims regarding outcome improvement should be interpreted cautiously and in relation to study design [5,26,27].

### 4.3. Language Mapping

Language mapping with nTMS is more complex and less standardized than motor mapping. Repetitive stimulation is applied while the patient performs language tasks, most commonly object naming, reading, or number counting. Errors are then classified and localized spatially. Unlike motor mapping, language mapping depends strongly on task design, baseline performance, error classification, stimulation frequency, patient cooperation, and lesion-related language reorganization [10,28,29].

Comparative studies with intraoperative direct cortical stimulation show that language nTMS can provide clinically useful information, but sensitivity, specificity, positive predictive value, and negative predictive value are highly variable across studies and cortical regions [5,28,29]. This variability limits the use of language nTMS as a standalone determinant of resection boundaries. Its current value is best understood as part of a multimodal risk stratification strategy that can inform the need for awake mapping, guide task selection, and help anticipate regions requiring intraoperative verification [4,5,10].

### 4.4. Integration with fMRI, Tractography, Neuronavigation, DCS, and IONM

In clinical practice, nTMS should be integrated with, rather than opposed to, other functional and structural methods. fMRI provides whole-brain task-related activation maps but relies on hemodynamic signals and can be affected by neurovascular uncoupling, mass effect, and poor task performance [6,7]. MEG can provide temporally precise functional information but is less widely available [6]. Diffusion tractography provides anatomical estimates of white-matter pathways but is not itself a functional test [1,4]. DCS and subcortical stimulation remain the intraoperative standard for real-time functional verification [1,2,5].

nTMS offers a stimulation-based preoperative map and can support planning of the craniotomy, approach trajectory, intraoperative monitoring strategy, and targeted DCS [4,9,10]. In systematic comparisons, the distance between motor sites identified by nTMS and DCS varies across studies, while language mapping shows wider ranges of sensitivity and specificity [5]. These findings support the clinical usefulness of nTMS while also emphasizing that preoperative maps must be interpreted in relation to intraoperative anatomy, brain shift, and stimulation findings [1,4,5].

A structured summary of selected clinical applications and their evidence status is presented in Table 1. The complementary roles of nTMS, fMRI, MEG, DCS/DES, and IONM are summarized in Table 2.

## 5. Broader Neurological and Therapeutic Applications: Contextual Overview

Broader neurological and therapeutic applications of TMS are important but extend beyond the primary neurosurgical focus of this review. Therefore, this section is intended as a contextual overview rather than a comprehensive disease-specific analysis. In neurological disorders such as amyotrophic lateral sclerosis, multiple sclerosis, epilepsy, and movement disorders, TMS-derived measures may provide information on corticospinal integrity, cortical excitability, inhibition, facilitation, and neuroplasticity [30,31,32,33]. However, these applications differ substantially in clinical validation, diagnostic specificity, and methodological standardization. TMS should generally be interpreted as an adjunctive neurophysiological tool rather than as a standalone diagnostic biomarker [30,31,32].

Therapeutic repetitive TMS (rTMS) has stronger evidence for some psychiatric indications, particularly major depressive disorder and obsessive–compulsive disorder, than for many neurological indications [34,35]. In stroke rehabilitation, chronic pain, tinnitus, Parkinson’s disease, and post-traumatic stress disorder, the evidence base is evolving but remains heterogeneous in terms of stimulation target, frequency, intensity, treatment duration, patient selection, and outcome measures [35,36,37]. The distinction between guideline-supported indications and investigational protocols is essential to avoid overgeneralization of therapeutic claims [35,37].

## 6. Safety Considerations in Neuro-Oncology and Patients at Increased Seizure Risk

TMS and nTMS are generally considered safe when performed according to established guidelines, but neuro-oncological patients require particular attention because their baseline seizure risk may be increased by cortical tumor involvement, peritumoral irritation, hemorrhage, edema, prior seizures, metabolic disturbances, sleep deprivation, or changes in antiseizure medication. Expert safety recommendations emphasize screening, individualized protocol selection, attention to stimulation parameters, and preparedness for seizure management [38].

Practical precautions before nTMS mapping should include assessment of seizure history, recent seizure control, current antiseizure medication regimen and adherence, sleep deprivation, alcohol or stimulant use, metabolic abnormalities, implanted devices, and other patient-specific risks. During mapping, unnecessary escalation of stimulation intensity and excessive train repetition should be avoided, especially in high-risk patients. Clinical observation should be continuous, and the team should have a predefined emergency plan, including safe positioning, airway precautions, trained staff, and access to rescue medication according to local protocols [38].

The absolute risk of TMS-induced seizure is low in contemporary practice, but it is not zero and varies with patient risk factors, stimulation paradigm, coil type, intensity, frequency, and train duration [38,39]. In our institutional experience with more than 90 patients undergoing nTMS-based preoperative mapping, no stimulation-induced epileptic seizure was observed. One examination was discontinued because of patient discomfort or malaise, without further adverse consequences. These observations support the feasibility and tolerability of the workflow in our setting but should not be interpreted as comparative safety data.

## 7. Limitations and Barriers to Clinical Implementation of nTMS

The main limitations of nTMS are methodological, anatomical, and system-level. First, preoperative-to-intraoperative spatial correspondence is limited by brain shift. After craniotomy and dural opening, cerebrospinal fluid loss, gravity, edema, tumor debulking, resection cavity formation, and brain relaxation can alter the relationship between preoperative functional maps and intraoperative anatomy [1,40]. Consequently, nTMS should be viewed as a perioperative planning tool rather than as a substitute for intraoperative functional verification with DCS, subcortical stimulation, and IONM [4,5].

Second, nTMS results depend on operator expertise, coil orientation, coil–cortex distance, stimulation intensity, target selection, and patient cooperation. Inter-operator variability may affect map reproducibility, particularly for language mapping, where task design and error classification are less standardized than in motor mapping. False positive and false negative responses may occur. Therefore, nTMS findings should be interpreted in a multimodal context and not as isolated binary markers of safe or unsafe cortex [5,10,28,29].

Third, stimulation depth and focality remain limited. Even with neuronavigation, the induced electric field is distributed over a cortical volume rather than a single point, and deeper or small cortical/subcortical structures cannot be mapped with the same precision as superficial motor representations [8,19,20].

Fourth, implementation requires dedicated equipment, high-quality MRI, trained personnel, integration with neuronavigation platforms, and multidisciplinary cooperation. These requirements limit scalability, particularly in smaller or non-academic centers [4,20].

Finally, reimbursement and national implementation frameworks remain important barriers in many healthcare systems, including Poland. Although the method provides clinically useful preoperative information, it is not uniformly recognized as a reimbursable diagnostic procedure. Wider adoption will require not only stronger evidence but also training standards, quality assurance, and health-policy recognition [1,4].

## 8. Clinical Workflow and Experience from Our Center

The Department of Neurosurgery at the University Clinical Center in Katowice implemented nTMS as part of routine preoperative planning for selected patients with intracranial tumors located in or near presumed eloquent cortical areas. This section is intended as a practical workflow illustration rather than as a formal outcomes study.

Patient selection is based on lesion location, expected proximity to motor or language-eloquent cortex, planned extent of resection, baseline neurological status, and the need for individualized risk stratification. All candidates undergo a high-resolution MRI acquired in a neuronavigation-compatible protocol. Imaging data are co-registered with the nTMS system, and the mapping plan is adapted to lesion location and clinical question.

**Figure 1:**
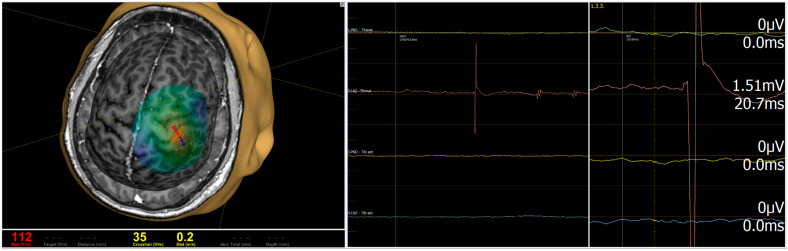
Single-pulse mapping of motor areas. Example of navigated transcranial magnetic stimulation (nTMS) motor mapping using the Nexstim NBS System 5, software version 5.2.4 (Nexstim Plc, Helsinki, Finland). The stimulation site (red vector) elicited a motor-evoked potential (MEP) in the contralateral upper limb. The color-coded activation map shows the cortical representation of the hand area overlaid on an individual MRI. The red vector indicates the stimulation site and coil orientation/trajectory. The colored traces on the right represent EMG/MEP recordings from monitored muscles.

Motor mapping begins with the identification of the motor hotspot and determination of MT. Single-pulse TMS is then used to localize cortical representations of clinically relevant muscles, with MEPs recorded using surface electromyography. An example of motor mapping obtained with the Nexstim NBS System 5, software version 5.2.4 (Nexstim Plc, Helsinki, Finland) is shown in Figure 1. The clinical value of such mapping lies not only in identifying the motor cortex, but also in documenting its spatial relationship to the lesion and in guiding decisions about craniotomy placement, surgical corridor, and intraoperative monitoring strategy.

Language mapping is performed when the lesion is located near presumed language regions or when the planned surgical strategy requires additional functional risk stratification. The patient first completes task training without stimulation to establish baseline performance. Repetitive stimulation is then applied while the patient performs tasks such as picture naming, reading, or number counting. Errors are classified and spatially documented. Because language mapping is more variable than motor mapping, its findings are interpreted together with clinical language status, handedness, imaging, fMRI when available, and the planned need for awake mapping.

**Figure 2:**
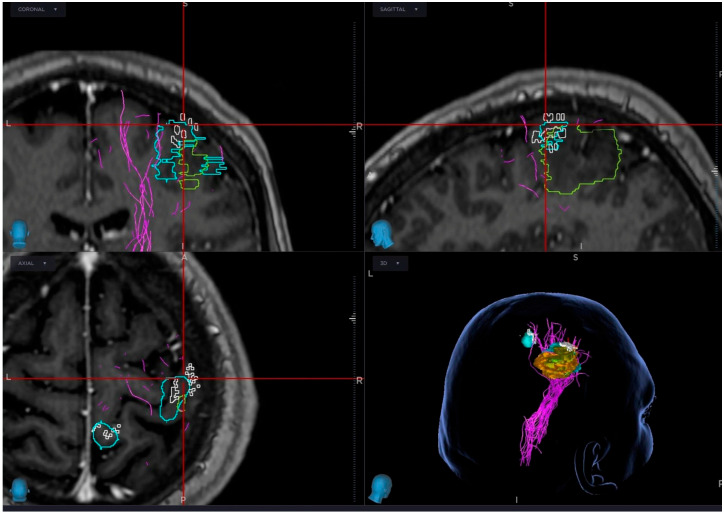
Coregistration of fMRI and nTMS functional maps. Integration of functional maps from fMRI and navigated TMS (nTMS) in neuronavigation system (StealthStation™ S8 planning station, StealthStation™ Application version 2.1.0, MNAV QS operating system version 2.0.3; Medtronic, Minneapolis, MN, USA). Functional motor areas (cyan—fMRI, white—nTMS) and tumor volume (green) are visualized with reconstructed corticospinal tractography (purple). Red lines indicate neuronavigation reference planes used for spatial orientation during surgical planning.

Functionally relevant motor and language points are exported into the neuronavigation system (StealthStation™ S8 planning station, StealthStation™ Application version 2.1.0, MNAV QS operating system version 2.0.3; Medtronic, Minneapolis, MN, USA), allowing visualization during surgical planning and, when appropriate, during the operation. Coregistration of nTMS with fMRI, tumor segmentation, and tractography is illustrated in Figure 2. This multimodal integration supports targeted craniotomy planning, trajectory selection, and identification of regions where intraoperative stimulation should be prioritized.

In practice, nTMS findings may support a more conservative strategy when functional points are close to the lesion, or may increase confidence in a more extensive resection when functionally relevant areas are displaced away from the surgical corridor. However, these decisions always require integration with intraoperative anatomy, DCS, subcortical stimulation, IONM, and surgical judgment.

To date, more than 90 patients have undergone nTMS-based mapping in our center as part of preoperative planning for intracranial lesions located in or near presumed eloquent cortical areas. During this period, no stimulation-induced epileptic seizure was observed. One examination was discontinued because of patient discomfort or malaise, without further adverse consequences. These observations support the practical feasibility of the workflow, but they should not be interpreted as comparative efficacy or safety data. A prospective institutional database with standardized endpoints would be required to formally analyze surgical decision changes, complication rates, postoperative functional outcomes, and concordance with intraoperative mapping.

## 9. Future Directions

Several research and implementation priorities emerge from the current evidence. First, multicenter prospective studies are needed to define standardized endpoints for nTMS-based mapping, including changes in surgical planning, concordance with DCS, extent of resection, postoperative functional outcome, seizure incidence, adverse events, and cost-effectiveness [5,26,27].

Second, motor and language mapping protocols require further standardization, particularly regarding stimulation intensity, coil orientation, task selection, error classification, and reporting of false positive and false negative results [5,10,28,29].

Third, nTMS should be increasingly integrated with advanced imaging, diffusion tractography, electric-field modeling, and automated analysis tools. Such integration may improve reproducibility and help move nTMS from point-based localization toward a broader model of patient-specific structure–function assessment [4,19,24].

Fourth, implementation studies are needed to determine how nTMS can be incorporated into national reimbursement systems, training curricula, and multidisciplinary neuro-oncology workflows [1,4,20].

## 10. Conclusions

nTMS is a clinically valuable noninvasive method for preoperative functional mapping, with the strongest current evidence in neurosurgical motor mapping for lesions located near eloquent cortex [4,26,27]. Its principal role is not to replace intraoperative functional verification, but to improve preoperative risk stratification, guide surgical planning, support multimodal neuronavigation, and facilitate targeted intraoperative mapping [4,5].

The broader diagnostic and therapeutic applications of TMS are important but heterogeneous, and they should be interpreted according to the level of evidence and clinical context. Future progress will depend on standardized protocols, prospective outcome data, integration with advanced imaging and modeling, and practical implementation frameworks. In centers managing complex neuro-oncological patients, nTMS has the potential to become an important component of personalized, functionally oriented neurosurgical care.

## Figures and Tables

**Table 1:** Clinical applications of TMS/nTMS and evidence status.

Application	Clinical Role	Evidence Status	Main Limitations
Preoperative motor mapping	Localization of motor cortex; risk stratification; craniotomy and approach planning	Relatively strongest evidence among neurosurgical nTMS applications; supported by observational studies and meta-analyses	Non-randomized evidence, brain shift, operator dependence, protocol heterogeneity
Preoperative language mapping	Adjunctive mapping before surgery near language regions; planning of awake mapping and task strategy	Promising but less standardized; variable agreement with DCS	Task dependence, error classification, false positives/negatives, patient cooperation
Tractography integration	nTMS-seeded corticospinal tract reconstruction and multimodal planning	Clinically useful in selected centers; evidence remains heterogeneous	Dependence on imaging protocol, tracking algorithm, edema, and brain shift
Neurological diagnostics	Adjunctive assessment of corticospinal integrity and cortical excitability	Disease-specific and supportive; not standalone diagnostic biomarker	Limited sensitivity/specificity data, heterogeneous protocols, biomarker competition
Therapeutic rTMS	Neuromodulation in selected psychiatric and neurological indications	Guideline-supported for some indications, exploratory for others	Protocol variability, patient selection, durability of effect, safety monitoring

**Table 2:** Comparison of selected functional mapping modalities in preoperative planning.

Method	Physiological Basis	Strengths	Limitations	Clinical Role
nTMS	Direct noninvasive cortical stimulation with EMG or task-based responses	Patient-specific, preoperative, stimulation-based, exportable to neuronavigation	Limited depth, coil orientation sensitivity, false positives/negatives, brain shift after opening	Preoperative functional map and risk stratification
fMRI	Task-related hemodynamic BOLD signal	Whole-brain coverage, widely used, noninvasive	Neurovascular uncoupling, task dependence, motion artifacts, indirect signal	Complementary functional imaging
MEG	Magnetic fields generated by neuronal activity	High temporal resolution, noninvasive	Limited availability, complex analysis, variable spatial localization	Adjunctive functional localization in selected centers
DCS/DES	Direct electrical stimulation of exposed cortex or subcortical pathways	Intraoperative reference standard; real-time functional testing	Invasive, limited to exposed area, affected by anesthesia and patient fatigue, seizure risk	Final intraoperative functional verification
IONM	Continuous or intermittent monitoring of functional pathways	Real-time warning during resection, especially motor pathways	Does not provide complete preoperative cortical map	Intraoperative safety monitoring

## Data Availability

No new data were generated or analyzed in this review. All data discussed are available in the cited literature.

## Abbreviations

The following abbreviations are used in this manuscript:

nTMS	Navigated transcranial magnetic stimulation
TMS	Transcranial magnetic stimulation
rTMS	Repetitive transcranial magnetic stimulation
IONM	Intraoperative neuromonitoring
fMRI	Functional magnetic resonance imaging
MEG	Magnetoencephalography
MRI	Magnetic resonance imaging
DCS	Direct cortical stimulation
DES	Direct electrical stimulation
EMG	Electromyography
MEP	Motor-evoked potential
MT	Motor threshold
BOLD	Blood oxygenation level-dependent
ALS	Amyotrophic lateral sclerosis
MS	Multiple sclerosis
PRISMA	Preferred Reporting Items for Systematic Reviews and Meta-Analyses
